# The Churches and Venereal Marriages

**Published:** 1917-12-15

**Authors:** 


### THE EDITOR'S LETTER-BOX.

### The Churches and Venereal Marriages.

*To the Editor of* THE HOSPITAL.

SIR,—In his comment on the article entitled "The Prophylaxis of Venereal Disease" Dr. G. Arbour Stephens expresses the opinion that the argument against the open proclamation of methods of medicinal prophylaxis stands or falls according as marriage is, or is not, regarded as a sacrament. That argument in brief was, that, as illicit sexual intercourse is essentially an anti-social proceeding, the medical profession ought not to be expected to take any steps to facilitate it, and I submit, with all respect to Dr. Stephens, that this position is not touched by any view which may be held as to the sacramental or non-sacramental character of marriage. This latter question may be of great importance in the theological sphere, and it is conceivable that, as a matter of doctrine or dogma, an organisation which believed itself, in the marriage ceremony, to be exercising sacerdotal functions, might claim the right to investigate the character and fitness of each of the proposed partners. But even granted such a position, how could this claim touch the demand that a knowledge of medicinal prophylactic agencies should be publicly and authoritatively put into circulation?

On the other hand, marriage may be regarded as a purely civil contract, and therefore subject to legal regulations and conditions; and amongst these may or may not be provisions relating to venereal disease in either of the partners and to the effect of such disease upon the validity or non-validity of the contract. In this country this non-sacramental view is probably the prevailing one, but its acceptance, it seems to me, has no relation to artificial prophylaxis either as a practice or as a policy. Dr. Stephens no doubt raises very important issues, but to intrude these into the argument of my paper is only to produce confusion. Whether he acted properly or improperly in communicating with the father of a syphilitic patient and with the priest who was to officiate at this patient's marriage; whether he believes marriage to be a sacrament or not a sacrament—in either case he can accept or challenge my conclusions. I shall, of course, be grateful for his support, but I cannot allow that my argument is affected by any views which he or anyone else may hold as to the theological nature and position of marriage.

—Yours, etc.,
THE WRITER OF THE ARTICLE.

